# Safety, tolerability, and impact on allergic inflammation of autologous *E.coli *autovaccine in the treatment of house dust mite asthma - a prospective open clinical trial

**DOI:** 10.1186/1472-6882-11-45

**Published:** 2011-06-03

**Authors:** Markus A Rose, Bianca Weigand, Ralf Schubert, Johannes Schulze, Stefan Zielen

**Affiliations:** 1 Dpt. of Allergy, Pulmonology, and Cystic Fibrosis, Children's and Adolescents' Hospital, Goethe University, Theodor Stern Kai 7, 60590 Frankfurt, Germany

**Keywords:** autovaccine, safety, tolerability, house dust mite allergy, asthma

## Abstract

**Background:**

Asthma is increasing worldwide and results from a complex immunological interaction between genetic susceptibility and environmental factors. Autovaccination with *E. coli *induces a strong TH-1 immune response, thus offering an option for the treatment of allergic diseases.

**Methods:**

Prospective open trial on safety, tolerability, and impact on allergic inflammation of an autologous *E.coli *autovaccine in intermittent or mild persistent house dust mite asthma. Determination of exhaled nitric monoxide (eNO) before and after bronchial mite challenge initially and after nine months of autovaccination.

**Results:**

In nine subjects and a total of 306 injections, we observed 101 episodes of local erythema (33.3%; median of maximal diameter 2.5 cm), 95 episodes of local swelling (31.1%; median of maximal diameter 3 cm), and 27 episodes of local pain (8.8%). Four subjects reported itching at the injection site with a total of 30 episodes (9.8%). Median eNO increase after autovaccination was significantly smaller (from 27.3 to 33.8 ppb; p = 0.334) compared to initial values (from 32.6 to 42.2 ppb; p = 0.046) (p = 0.034). We observed no serious adverse events. All organ functions (inclusive electrocardiogramm) and laboratory testing of the blood (clinical chemistry, hematology) and the urine (screening test, Β-microglobuline) were within normal limits. Vital signs undulated within the physiological variability.

**Conclusion:**

The administration of autologous autovacine for the treatment of house dust mite asthma resulted in a reduction of the eNO increase upon bronchial mite challenge. In nine subjects and 306 injections, only a few mild local reactions and no systemic severe adverse events were observed.

**Trial registration:**

**EudraCT Nr**. 2005-005534-12

**ClinicalTrials.gov ID **NCT00677209

## Background

Asthma is increasing worldwide and results from a complex interaction between genetic susceptibility and environmental factors. Autovaccination with *E. coli *induces in animal models and in human studies a strong TH-1 immune response. In allergic individuals, this immunization can cause a shift from TH-2- to TH-1- dominated immune response, and has the potential to work as an unspecific immunotherapy, evoking immunotolerance [1].

Allergic diseases such as rhinoconjunctivitis or bronchial asthma are a major burden of disease, requiring evidence-based therapy concepts. So far, treatment mainly encompasses antiallergics, anti-inflammatory drugs (e.g., steroids), or bronchodilators. The only causative therapeutic option is the specific immunotherapy (SIT), administering the patient the relevant allergen in increasing doses [2], thus inducing immunotolerance.

In immunotolerance towards house dust mite, an allergenspecific T-cell suppression directed against the house dust mite allergen Der p1 is observed. In allergics, this TH1 (IFN-y)-immunresponse is reduced, with a shift towards a TH2-dominated (IL-5, IL-13) immune pattern. Neutralization tests of cytokine activity revealed that the T-cell-suppression in specific immunotherapy and immunotolerance against mucosal antigens is induced by IL-10 and TGF-ß. In addition, specific immunotherapy results in an antigenspecific suppressive activity in CD4(+) CD25(+)-T-cells of allergic subjects. In conclusion, during SIT a shift towards a regulatory/suppressor-T-Zell-response (TH-1) can be observed, which is a key phenomenon for the induction of immunotolerance. One must admit, that depending on the age and individual features of house dust mite allergics, SIT is of heterogeneous efficiency [3], demanding even more therapeutic alternatives.

The determination of exhaled nitric oxide (eNO) is an established tool to monitor bronchial inflammation. We used the increase of eNO following specific bronchial provocation with house dust mite extract before and after the autovaccine treatment as a surrogate for the autovaccine's potential impact on allergic asthma. In the last decades, bacterial autovaccines have been used in general medicine worldwide [4-12]. Despite their broad use for many indications (e.g., chronic infections), no studies according to good clinical practice have been performed. This is the first prospective study to investigate the safety, tolerability, and impact on allergic inflammation of an autologous *E. coli *autovaccine in patients with house dust mite allergy and bronchial asthma.

## Methods

### Participants

In our investigator-initiated prospective study, a total of nine subjects were recruited from our walk-in clinic. Included were clinically healthy 18-50 year old asthmatics (GINA 0-1°; episodic bronchial obstruction, on-demand therapy only) with skin prick test and bronchial challenge positive for house dust mite (FEV1 decrease ≥ 20%). Exclusion criteria were a history of intolerance towards the ingredients of the autovaccine, asthma ≥GINA II°, other chronic infections/diseases, pregnancy, intermittent treatment with systemic steroids or permanent treatment with inhalative steroids, other immunmodulation or -suppression, any known substance abuse, the incapability to understand and perform the study, and tobacco smoking. Women of childbearing age were instructed to perform contraception. All participants supplied written informed consent prior to the study. Human experimentation guidelines of Good Clinical Practice, the German Drug Act and the declaration of Helsinki/Hong Kong were followed in the conduct of clinical research. The study had been approved by the ethical committee of the University of Frankfurt.

### Study design and assessment

**Figure 1:**
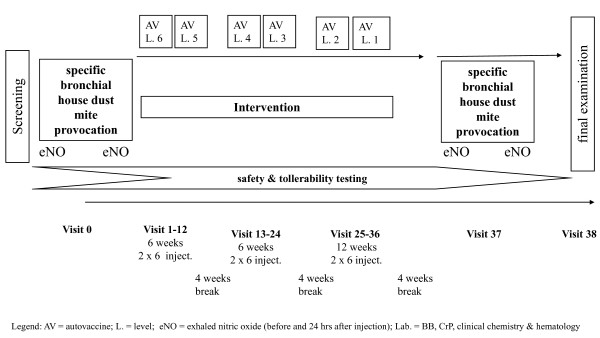
**Autologous *E. coli *autovaccine in house dust mite asthma. **Figure 1 illustrates the study design of the trial

Included participants underwent a 37 week protocol with autologous *E. coli *autovaccine being subcutaneously applied in incremental doses as established in clinical routine practise (see figure 1). At the beginning of every visit, body temperature was determined and a physical examination performed. Between visits 12 and 13, 24 and 25, and 36 and 37 a 4-week-break was included to facilitate immunological conditioning. At every visit, a screening for kidney function was performed (Combur-Test^®^, Fa. Roche Diagnostics; detection of leucocytes, nitric, pH, protein, glucose, ketones, urobilinogen, bilirubin, blood, and hemoglobine). At visit 0, 18 (-in the middle of the intervention), and 38, additionally beta-microglobuline was determined as tubular marker. At visit 0, 18, and 38, also an electrocardiogram was performed. After every injection, participants were monitored clinically (heart rate, body temperature, and blood pressure) every 30 minutes for two hours. Measuring of body temperature and peak flow was continued at home until 12 hours after the injection. Adverse events were recorded with the trial diary. At visit 0 and 37 (after the intervention), specific bronchial provocation and measurement of eNO were performed to assess the potential impact of the autovaccine (figure 1). After another 24 hours and on visit 38, study participants underwent a final examination.

### Instruments

Specific bronchial challenge was delivered via a medic aid nebulizer and the aerosol provocation system APS powered by compressed air (all by Viasys Inc., Wuerzburg/Germany). Exhaled nitric oxide (eNO) as a surrogate for bronchial inflammation was determined using a NO-analyzer (NIOX^®^, Aerocrine Inc., Stockholm/Sweden) according to the criteria of the American Thoracic Society [13], before starting any inhalation and 24 hrs after the challenge. In order to control for intrasubject variability, the mean values of three consecutive measurements were taken.

### Therapeutic intervention

The autovaccine produced by Symbiovaccin, Herborn/Germany is a patient-specific *E.coli *autovaccine, which has been used for decades in many European countries. Apathogen *E.coli *is isolated from the patient's feces, processed according to a standardized procedure and twice heat-inactivated (75°C) over two hours [14] with level 1 containing 1 × 10^8 ^- 1 × 10^9 ^units germs/ml, level 2 containing 1 × 10^7 ^- 1 × 10^8 ^units germs/ml, level 3 containing 1 × 10^6 ^- 1 × 10^7 ^units germs/ml, level 4 containing 1 × 10^5 ^- 1 × 10^6 ^units germs/ml, level 5 containing 1 × 10^4 ^- 1 × 10^5 ^units germs/ml, level 6 containing 1 × 10^3 ^- 1 × 10^4 ^units germs/ml. Additionally, isotonic saline solution (sterile, free from pyrogenes, 996.5 μl) and Phenol liquefactum 3.5 mg as preservative. The immunization schedule starts with level 6 and concentrations increase up to level 1; in case of intolerance towards the autovaccine, increase of doses was slowed down, or concentrations went back to the last tolerated step for two weeks. If this did not result in tolerance of the autovaccine, or severe side effects occurred, the participant would have dropped out of the study.

### Outcomes and laboratory testing

Primary outcome measures were the occurrence of adverse events, the tolerability of the intervention, and safety laboratory testing including clinical chemistry (CrP, GOT, GPT, y-GT, aP, creatinine, and urea), hemostasis (Quick, PTT, and fibrinogen), hematology (red and white blood cell count, thrombocytes), markers of kidney function (urine-stix, beta-microglobuline), and the electrocardiogram. These blood and urine testings were performed on each visit. Secondary outcome parameter were the impact of the autovaccine treatment on exhalative nitric oxide as a marker for bronchial inflammation, and the impact on markers of allergy (eosinophilic cationic protein (ECP), total IgE, and specific IgE against house dust mite *Der. p1*. and *Der. far*., which were determined at the beginning of the study (visit 0) and after the intervention (visit 37).

### Statistical analysis

Since this study was a pilot study investigating safety and tolerability, analysis was mainly based on descriptive approaches. In the absence of normally distributed values, medians and standard deviations (SDs) were calculated for every visit. Also the analysis of exhalative nitric oxide values was performed by non-parametric tests (Mann-Whitney, Wilcoxon). A probability (p)-level < 0.05 was considered as statistically significant.

## Results

### Baseline characteristics of participants

A total of 15 subjects were screened, of which nine (female:male = 6:3) could be included into the study. The other six subjects considered the protocol as too time consuming. Eight subjects completed the study with 100% adherence to all visits; one subject (No. 2) dropped out due to urticaria, which had also been observed before the study started (table 1). All participants used short-acting betamimetics on demand without change of the dosage or frequency in the course of the study. Measuring of peak flow revealed no negative acute impact of the autovaccine application on the lung function.

**Table 1:** Features of the study population

subject **No**.	gender	age [years]	height [cm]	weight [kg]	BMI	FEV1 [%]	therapy	Asthma severity
1	female	27	161	52	20.1	98.0	BoD	<1/w

2	female	27	170	64	22.1	104.0	BoD	<1/w

3	female	29	170	70	24.2	94.0	-	<1/w

4	female	28	166	54	19.6	109.0	-	<1/w

5	male	22	187	85	24.3	127.0	-	<1/w

6	male	34	176	80	25.8	118.0	-	<1/w

7	female	27	166	59	21.4	82.7	BoD	<1/w

8	female	31	180	66	20.4	97.0	-	<1/w

9	female	49	163	65	24.4	99.0	BoD	<1/w

BoD = betamimetic inhalation on demand; <1/w = less than one exacerbation/week & no limitation of activities

### Local reactions

**Erythema **at the injection site as an expression of a local immune reaction was observed in every patient, starting from different dosage levels (see table 2). In a total of 306 injections, we observed 101 episodes of erythema (33%) with a median of 12/36 injections per patient (range 1-26), with a median maximal diameter of 2.5 cm (range 2-11 cm).

**Table 2:** Local reactions after autologous *E.coli *autovaccine

Subject	Erythema	Induration	Local pain*	Itch
**No. 1**	V32 →	V32 →	V32 →	-

**No. 2**	V16 →	-	-	-

**No. 3**	V22 →	V23 →	V22	-

**No. 4**	V25 →	V25 →	-	-

**No. 5**	V29 →	V29 →	-	-

**No. 6**	V26 →	V26 →	-	V26 →

**No. 7**	V22 →	V22 →	V22	V31 →

**No. 8**	V13 →	V13 →	-	V8/16/25; V27 →

**No. 9**	V9 →	V9 →	V17 →-	V9

**episodes total No**. (per 306 injections)	101 (33%)	95 (31%)	27 (8.8%)	30 (9.8%)

**episodes/subject **[median No., range]	12 (1-26)	10 (0-25)	n.a.	n.a.

**average size **[cm, range]	2.5 (2-11)	3.0 (0-11)	n.a.	n.a.

V32 → = after visit 32 and every following visit; V22 = after visit 22; * = mild in character; n.a. = not applicable

**Swelling **at the injection site occurred in eight of nine subjects, starting from different dosage levels. Among 306 injections, we observed a total of 95 episodes of swelling (31%). There was a median of 10 swellings/36 injections per patient (range 0-25), with a median of the maximal diameter of 3 cm (range 0-11 cm).

**Local pain **was reported by four of nine patients. In contrast to erythema and induration, which were observed repeatedly after reaching the reaction level, local pain recurred only in two subjects, while two other subjects reported local pain only once each (probably by accidental irritation of a skin nerve by the needle). Among 306 injections, 27 episodes of local pain (8.8%) were reported, among them 20 episodes in one subject and 1 episode each in two subjects in the middle of the study, without further reporting in the continuation of the study.

**Itching **at the injection site was reported by four of nine subjects. It occurred in 30/306 injections (9.8%).

**Urticaria **exclusively occurred in subject No. 2 starting from visit 16 and upon mechanical irritation of the skin, disappearing with dose-reduction and reoccurring after increasing concentrations of the autovaccine. The patient reported similar episodes of urticaria already in the past. Despite the mild character of the complaints, the patient was excluded from the study.

In the course of the study, one female subject experienced a urinary tract infection, which was considered as not causally related. We observed no other **serious adverse events**. All organ functions (inclusive electrocardiogramm) and laboratory testing of the blood (clinical chemistry, hematology) and the urine (screening test, B-microglobuline) were within normal limits.

**Vital signs **undulated within the physiological variability (see table 3). The **peak-flow **measurement following each visit revealed no negative impact of the autovaccine on acute lung function.

**Table 3:** Vital signs after autologous *E.coli *autovaccine application

	**Heartbeat/min**.					Body temperature [°C]				
	**0'**	**30'**	**60'**	**90'**	**120'**	**0'**	**30'**	**60'**	**90'**	**120'**

**Subject No. 1**										

median	75.0	72.0	74.0	75.0	72.0	36.4	36.2	36.2	36.4	36.4

SD	6.03	6.55	5.31	5.15	5.27	0.68	0.69	0.64	0.62	0.57

**Subject No. 2**										

median	86.0	83.5	83.0	80.0	77.5	36.8	37.0	37.0	36.9	36.8

SD	6.55	12.2	11.2	12.5	9.72	0.32	0.309	0.207	0.2	0.19

**Subject No. 3**										

median	64.0	64.0	62.0	58.0	59.0	36.2	36.3	36.3	36.4	36.2

SD	8.88	5.4	5.60	8.16	5.66	0.34	0.30	0.31	0.38	0.27

**Subject No. 4**										

median	64	80	78.5	80	78	36.4	36.5	36.7	36.6	36.7

SD	16.0	12.5	13.2	7.8	8.48	0.29	0.37	0.54	0.33	0.29

**Subject No. 5**										

median	76	69.5	70.5	69.0	66.0	36.2	36.3	36.2	36.4	36.3

SD	10.2	12.6	11.7	11.8	7.87	0.20	0.30	0.19	0.26	0.16

**Subject No. 6**										

median	79.0	76.0	74.5	74.0	73.5	36.2	36.20	36.2	36.3	36.3

SD	11.0	7.94	5.82	4.63	5.17	0.22	0.26	0.30	0.27	0.31

**Subject No. 7**										

median	75.0	75.0	68.5	74.5	73.0	36.4	36.2	36.2	36.1	36.2

SD	4.90	5.97	7.87	5.72	5.43	0.33	0.29	0.29	0.21	0.37

**Subject No. 8**										

median	80	80	80	79.5	80	36.1	36.1	36.1	36.1	36.1

SD	6.35	6.16	6.65	7.23	5.81	0.05	0.05	0.05	0.04	0.05

**Subject No. 9**										

median	72	68	68	68	68	36.1	36.1	36.1	36.1	36.1

SD	9.70	10.1	9.51	8.35	8.51	0.05	0.05	0.06	0.04	0.04

SD = standard deviation

#### Blood and urine testing, ECG

The markers of clinical chemistry (CrP, GOT, GPT, y-GT, aP, creatinine, and urea), hemostasis (Quick, PTT, and fibrinogen), hematology (red and white blood cell count, thrombocytes), and of kidney function (urine-stix, beta-microglobuline) undulated within the physiological and laboratory variability and indicated no negative impact of the autovaccine treatment (tables 4, 5). The electrocardiogram before, during and after autovaccine treatment remained unchanged.

**Table 4:** Laboratory testing - haematology

subject		Hb	Hkt	MCV	Erys	Leuc	Thromb	Lymph	Monos	Neutr	Eos	Basos
No. 1	median	11.7	37	84.5	4.48	6.6	364	36.8	5.2	53.5	3.6	0.7
	
	SD	0.65	1.89	1.60	0.18	1.10	31.2	6.08	0.87	6.49	1.14	0.37

No.2	median	12.3	37.9	87.1	4.3	5.6	328.6	29.6	4.1	48.3	1.8	0.4
	
	SD	5.7	17.6	40.1	2.0	2.7	15.5	14.4	2.0	22.8	1.3	0.3

No.3	median	13.2	37.6	90.8	4.13	5.87	226.5	36.4	4	54.9	4.4	0.3
	
	SD	0.54	1.32	1.20	0.17	0.84	21.2	5.95	0.81	6.84	1.16	0.20

No.4	median	14.1	41.75	87.4	4.78	5.62	209	42.5	4.4	49.7	3.1	0.6
	
	SD	0.40	1.31	0.84	0.12	0.86	15.1	5.28	0.64	5.66	0.83	0.33

No.5	median	14.3	41.5	86.7	4.81	4.62	264	30.9	9	55	4.8	0.8
	
	SD	0.67	1.56	1.65	0.15	0.92	14.5	4.69	1.27	5.77	1.57	0.60

No.6	median	15.9	45.9	85.9	5.35	5.79	207	44.1	5.7	40.4	8	0.6
	
	SD	0.49	1.35	0.74	0.17	1.11	12.1	7.00	1.10	7.32	2.25	0.33

No.7	median	12.1	35.9	86.6	4.16	3.96	236	38.5	5.4	54	1.5	0.3
	
	SD	0.48	1.49	1.45	0.15	0.62	23.7	5.96	0.97	6.68	0.58	0.31

No.8	median	12.8	38.1	89.3	29.6	33.2	4.29	7.24	329	37.0	4.9	55.1
	
	SD	0.60	1.79	1.48	0.65	0.41	0.23	1.39	23.1	5.67	0.87	6.17

No.9	median	12.2	35.6	88.3	30.4	34.5	4.04	5.1	303	36.5	6.0	55.4
	
	SD	0.47	2.07	0.83	0.45	0.64	0.14	0.72	23.7	4.59	0.92	5.23

Hb = haemoglobine [g/dl]; MCV = mean corpuscular volume [fl]; Erys = eythrocytes [10^6^/ml]; Leuc = leucozytes [1000/ml]; Lymph = lymphocytes [%]; Monos = monocytic granulocytes [%]; Neutr = neutrophilic granulocytes [%]; Eos = eosinophilic granulocytes [%]; Basos = basophilic granulocytes [%]; SD = standard deviation

**Table 5:** Laboratory testing - clinical chemistry

subject		Quick	PTT	Fib	GOT	GGT	GPT	AP	CRP	Krea	Bun	ß2 Mikrog
No. 1	median	87	33	247	20	20	12	22	0.09	0.69	20	1.3
	
	SD	5.98	1.44	30.3	3.56	2.87	4.69	4.02	0.05	0.07	3.92	0.13

No.2	median	82.9	31.8	219.0	16.7	18.4	8.2	17.7	0.1	0.6	16.1	1.2
	
	SD	3.84	14.9	12.6	8.5	8.8	5.2	8.9	0.0	0.3	7.8	0.5

No.3	median	90	35	235	22	12	15	35	0.03	1.06	29.5	1.4
	
	SD	4.61	1.43	46.1	2.70	2.02	2.54	4.59	0.36	0.07	5.30	0.13

No.4	median	96.5	30	275.5	25	21	18	52	0.06	0.79	24	1.2
	
	SD	5.12	1.47	38.1	3.10	2.36	5.02	5.70	0.20	0.07	3.93	0.08

No.5	median	92	33	232	27	15	22	66	0.04	0.99	29	1.7
	
	SD	4.71	1.04	41.9	5.20	1.08	4.68	4.39	0.12	0.09	3.12	0.15

No.6	median	108	33	283.5	20	30	18	58	0.05	0.93	28	1.3
	
	SD	4.64	1.81	44.2	2.66	1.73	2.26	7.52	0.09	0.05	5.64	0.13

No.7	median	91	34	293	21	42	18	49	0.21	0.74	27	1.75
	
	SD	3.91	0.94	27.8	1.92	3.81	2.47	6.21	0.15	0.07	3.18	0.13

No.8	median	110	31	244	19	39	20	83	0.07	1.01	33	1.6
	
	SD	6.99	1.92	23.8	2.31	3.89	3.26	6.58	0.22	0.07	5.11	0.09

No.9	median	88	33	220	21	11	14	34	0.05	0.63	24	1.3
	
	SD	4.52	1.15	23.2	1.77	2.09	1.91	2.08	0.06	0.07	3.99	0.097

Quick = thromboplastin time [%]; PTT = partial thromboplastin time [sec.]; Fib = fibrinogen [mg/dl]; GOT = aspartat aminotransferase [U/l]; yGT = gamma glutamyltransferase [U/l]; GPT = alanin aminotransferase [U/l]; AP = alkaline phosphatase [U/ml]; CrP = C-reactive protein [mg/dl]; Krea = creatinine [mg/dl]; Bun = urea [mg/dl]; ß2 Mikrogl = ß2 microglobuline [mg/l]; SD = standard deviation

#### Markers of Allergy

The determination of allergy markers before (visit 0) and after autologous autovaccine treatment (visit 37) revealed a reduction without reaching statistical significance. Median levels (Q25, Q75) of the eosinophilic cationic protein (ECP) decreased from 22.7 (9.45, 36.9) μg/ml to 17.7 (9.53, 21.8) μg/ml (p = 0.144). Median levels of IgE decreased from 206 (47, 261) IU/ml to 127 (81, 152) IU/ml (p = 0.490). Specific IgE against *Der. p1*. decreased from 30.5 (8.96-46.1) to 28.3 (12.3-40.9) IU/ml (p = 0.468), against *Der. far*. from 28.1 (6.67. 43,1) to 23.1 (9.93, 45.9) IU/ml (p = 0.489).

#### Exhaled Nitric Oxide

**Figure 2:**
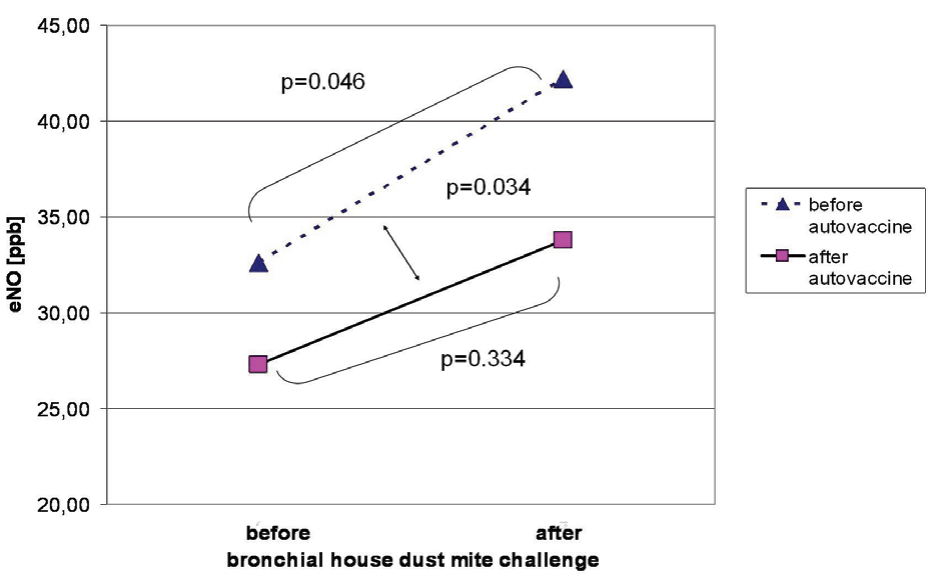
**Impact of E.coli autovaccine on exhaled NO as marker of bronchial inflammation in house dust mite allergic asthma. **Figure 2 illustrates the increase of exhaled nitric oxide under specific bronchial challenge before and after autovaccine application

Median (25^th ^and 75^th ^quartile) values of exhaled nitric oxide at visit 0 were 32.6 (20.8-37.4) before house dust mite challenge, and 42.2 (28.2-75.8) ppb, corresponding with a 1.36-fold increase (p = 0.046). After the autovaccine treatment at visit 37, basal median eNO was 27.3 (13.4-51.0) ppb and increased within 24 hrs to 33.8 (14.1-56.0) ppb, corresponding to a 1.13-fold increase (p = 0.334). Over all, the eNO increase before the autovaccine treatment was significantly higher than after the intervention (p = 0.034; figure 2).

## Discussion

As early as in the beginning of the last century, a staphylococci autovaccine had been developed in England [15]. The success of antibiotics made this mile stone disappear, but in the last decades, especially Eastern European groups reported the successful usage of autovaccine in the treatment of severe acne vulgaris, chronic sinusitis, otitis media etc. [6-8].

In this first prospective clinical trial, which was focused on safety and tolerability, we also observed a mild but significant reduction of the exhaled nitric oxide as a surrogate marker for bronchial inflammation. As expected, in the beginning of the study values of eNO increased significantly after bronchial mite challenge. After 37 weeks and autovaccine treatment, eNO-values were lower before and after the challenge; also the increase due to the provocation was significantly lower. This goes with Halasa and coworkers from the University of Stettin, who have been treating bronchial asthma with autovaccine over decades [16].

In the autovaccine treatment, a typical acute-phase reaction with a rapid inflammatory response can be observed with the deliberation of various mediators [17]. Besides this unspecific phenomenon, also specific responses can be induced. Following an autovaccine derived from Propionibacterium acnes, specific antibodies against structural antigens of *P. acnes *could be proven [6].

Also an activation of antigen-specific T-helper cell-subpopulations could be observed [7]. The underlying immunological mechanisms could be illuminated in animal models. C57BL/6-mice were sensitized with ovalbumin, resulting in bronchial hyperresponsiveness. Then, they were treated with autovaccine, and following an aerosolprovocation, bronchial hyperreactivity was found to be blocked. Autopsy revealed a significant suppression of specific IgG-1-antibodies, and a reduced bronchial inflammation as illustrated by a reduction of eosinophils and lymphocytes in the bronchoalveolar lavage. This was accompanied by an increase of anti-inflammatory IL-10 and IFN-y [18].

Autovaccines have been used for more than a century in veterinary and human medicine worldwide. Established indications are chronic infections of the airways and associated sinuses, the skin (furunculosis), and the bones (osteomyelitis) [e.g., 9]. A Tschec study demonstrated in 33 patients with chronic upper airway infections a therapeutic success of the autovaccine, persisting over three years of follow-up [10].

Another trial from Poland prospectively treated 292 children suffering from sinusitis showed a success of autologous autovaccine in 88% of the patients [7]. A Russian group successfully treated sinusitis ethmoidalis with autovaccine [9]. In Germany, a study demonstrated the autovaccine's usefulness in cows suffering from genital infections [11].

So far, only one GCP-conform clinical trial has been performed, treating 78 adult patients suffering from various infections (32% skin, 42% airways) with *E. coli *autovaccine and demonstrating good tolerability. Within this study, blood of patients underwent an *ex vivo *stimulation of leucocytes with autovaccine, inducing a decrease of the cytokines GM-CSF and INF-y, while IL-1ß and IL-6 increased [19].

Patients suffering from allergic asthma typically react upon bronchial challenge with their specific allergen with a deterioration of their lung function and with clinical complaints (e.g., cough). We could demonstrate in an antecedent study the protective effect of anti-inflammatory polyunsaturated fatty acids as to bronchial response upon a bronchial house dust mite challenge [19]. We postulate that also the anti-inflammatory effect of autovaccine can be investigated analogously. In addition, the determination of eNO is an established surrogate marker for airway inflammation.

Just like in other immunotherapies, local (at the injection site) and systemical side effects can occur [20]. In addition, with incremental dose often an individual "reaction threshold" is observed, showing profound reactions at the injection site within 24 hours, in rare cases also systemic reactions (irritation, pain, and an increase of body temperature). No other severe side effects have been observed so far in clinical routine. Autologous autovaccine had no negative acute impact on the lung function as proven by peak flow measurements after the application. In contrast, we observed mild positive effects on markers of allergic inflammation (eNO, ECP, IgE). Our study is the first to prospectively investigate a substance which has been used for decades in complementary medicinal practice. We estimated risks and side effects to be comparable with the established specific immunotherapy, but in contrast to SIT, we did not expect allergic reactions upon a specific allergen [21]. Our study demonstrated a good tolerability of the autologous autovaccine in the treatment of mild to moderate bronchial asthma in the context of a house dust mite allergy. We found a low number of local reactions and - with the exception of one urticaria factitia (urticaria upon mechanical pressure) no systemic side effects. The affected person had already intermittently experienced urticaria ahead of the study. Nonetheless, since the urticaria disappeared in parallel with the withdrawal of the study medication and reoccurred with the re-challenge, we judged it as potentially causal related. This goes with the observation of urticaria occurring as an autoimmune-phenomenon. Since the aim of immunotherapy is a shift towards a TH-1 dominated immune response, autoimmune phenomena, which are perceived as TH1-overregulations, might occur in this context. On the other hand, it might have been the spontaneous and natural course of the urticaria in an individual with recurrent urticaria.

We observed the individually varying "threshold of reaction" as described by the manufacturer and many applying physicians only in very mild forms. In a few cases and intermittently, the injection was perceived as painful, what we interpreted as mechanical irritation due to the injection needle, and independent of the autovaccine. We observed - as expected- mild local reactions as an expression of immunological activation, but no classical allergic or anaphylactic reactions, with the restriction, that due to the limited number of applications we might have missed such relatively rare events. Thus, we cannot come to a conclusive judgment of this aspect of drug safety. As with all studies, we relied on accurate reporting of symptoms and complaints and accepted the limitations of this approach as to our primary endpoints. Also the small number of the analysed group might have resulted in type 2 errors.

Over all, our study underlines the experiences with autovaccine application from daily medical practice. It seems that its tolerability is even superior to the established conventional allergen specific immunotherapy.

## Conclusions

In patients with house dust mite asthma, an incremental schedule of autologous autovaccine was safe and well-tolerated. Although specific immunotherapy (SIT) already exists, autologous autovaccine might be an interesting option in multiple allergies, where SIT is not working. The observed impact on bronchial inflammation as expressed by a reduced increase of exhaled nitric oxide will be the issue of a larger placebo-controlled study.

## Competing interests

The study was partly funded by Symbiopharm Inc., Herborn, Germany. MAR and SZ received lecture fees from Symbiopharm.

## Authors' contributions

We hereby declare that every author and co-author substantially contributed to the work. MR developed the study design, performed data analysis and wrote the manuscript. BW performed the whole clinical part of the study and collected data. RS made the whole laboratory testing, JS performed the bronchial testing, SZ developed the study and supervised the performance. All Authors and co-authors carefully revised and approved the manuscript.

## Pre-publication history

The pre-publication history for this paper can be accessed here:

http://www.biomedcentral.com/1472-6882/11/45/prepub
